# Elimination of Aberrant DRG Circuitries in Sema3A Mutant Mice Leads to Extensive Neuronal Deficits

**DOI:** 10.1371/journal.pone.0070085

**Published:** 2013-07-26

**Authors:** Ayal Ben-Zvi, Sahar Sweetat, Oded Behar

**Affiliations:** Department of Developmental Biology and Cancer Research, IMRIC, Faculty of Medicine, The Hebrew University, Jerusalem, Israel; McGill University, Canada

## Abstract

Axon guidance molecules determine the pattern of neuronal circuits. Accuracy of the process is ensured by unknown mechanisms that correct early guidance errors. Since the time frame of error correction in Sema3A null mice partly overlaps with the period of naturally occurring cell death in dorsal root ganglia (DRG) development, we tested the hypothesis that apoptosis of misguided neurons enables error correction. We crossed BAX null mice, in which DRG apoptosis is blocked, with Sema3A null mice to induce errors. Analyses of these double-null mouse embryos showed that the elimination of abnormal projections is not blocked in the absence of BAX. Surprisingly however, there are fewer surviving neurons in Sema3A null or Sema3A/BAX double-null newborn mice than in wild-type mice. These results suggest that guidance errors are corrected by a BAX-independent cell death mechanism. Thus, aberrant axonal guidance may lead to reductions in neuronal numbers to suboptimal levels, perhaps increasing the likelihood of neuropathological consequences later in life.

## Introduction

Nervous system function is critically dependent on highly stereotypic and precise patterns of neuronal connectivity established during embryogenesis. The formation of these connections depends on the ability of axons to probe their environment and selectively navigate to their targets in an accurate manner [1]. Axon guidance is accomplished by repellent and attractant guidance molecules such as semaphorins or netrins [2]. Analyses of the Semaphorin 3A (Sema3A) knockout mouse revealed a significant level of abnormal guidance errors at embryonic days (E) 11–13 [3]. Surprisingly, these extensive abnormalities largely disappear by E15.5 [4]. Similar findings were reported for Netrin-1 null mice, which also exhibit DRG axon guidance errors at E11.5 [5]. As is the case for Sema3A null mice, the navigation errors disappear by E13.5 [5]. These studies demonstrate the existence of an “oversight” mechanism capable of correcting errors in axonal path-finding. The mechanism responsible for this robust correction is not known.

**Figure 1:**
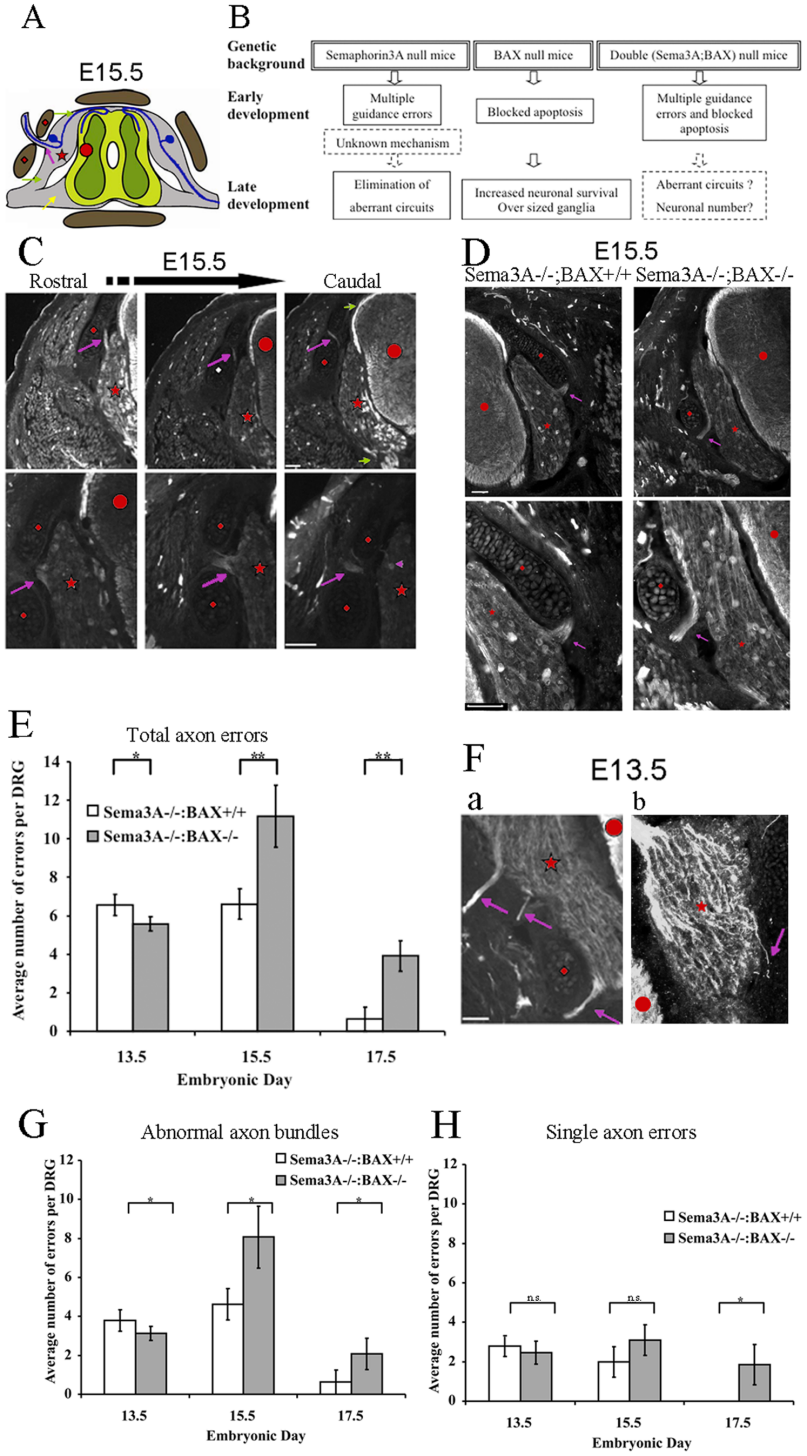
Abnormal axon projection elimination is not blocked in the absence of BAX. **A:** Illustrated cross section of the lumbar spinal cord at E15.5 used to analyze abnormal axon projections. Spinal cord in the middle (red circle) with DRG on both sides (red star) is surrounded by pre-cartilage primordial (red rhombus). The left side portrays an abnormal circuit – pseudo-uni-bipolar neurons within the DRG (blue) send branches and enter the spinal cord at the dorsal root entry (green arrow). A second branch crosses abnormally through the pre-cartilage primordial (purple arrow) instead of using the correct path through the ventral root (green arrow – same marking shapes are used hereafter). The right side portrays a normal circuit (note that the ventral root contains the ventral nerve of the DRG and the motor nerve exiting the spinal cord ventrally). **B:** Experimental design. Mouse genetics used to test the role of apoptosis in the elimination of aberrant circuits. C**:** Abnormal axon projections in three adjacent cross-sections of DRG from a Sema3A^−/−^ embryo (E15.5). Note the normal dorsal and ventral roots (short green arrows, upper right image). Abnormal projections were scored only when actual exit from the DRG was noticeable (arrows in upper and lower left images). Serial sections were used to identify *bona fide* errors. Low magnification in upper panel and high magnification of a different error in lower panel; note that single axon errors could be detected at this magnification (lower right image, purple arrowhead). All scale bars 50 µm. **D:** Similar axon errors are detected in Sema3A null mice and Sema3A:BAX double null mice. Abnormal axon projections in Sema3A^−/−^:BAX^+/+^ mice (left) or Sema3A^−/−^:BAX^−/−^ mutant mice (right). The abnormalities are similar in both cases. A representative image of lumbar DRG cross section is shown (low magnification, upper panel) and (higher magnification, lower panel) of the same abnormal projections (anti-neurofilament immunostaining). At E15.5, these DRGs of Sema3A^−/−^:BAX^+/+^ and Sema3A^−/−^: BAX^−/−^ embryos exhibit abnormal axon bundles (purple arrow). Spinal cord is marked with a red circle, the pre-cartilage primordial marked with a red rhombus, and the DRG is marked with a red star. All scale bars are 50 µm. **E:** Analysis of temporal error occurrence. Error rate over time in Sema3A^−/^: BAX^+/+^ embryos compared to error rate in the compound Sema3A^−/−^:BAX^−/−^ embryos. Absence of BAX results in accumulation of errors between E13.5 and E15.5. Massive error elimination is apparent in both genetic backgrounds at E17.5 (n = 30 DRG per genotype in each of the three embryonic ages, E13.5 P = 0.037, E15.5 P = 0.05, and E17.5 P = 0.0042). **Fa,b:** Representative images of E13.5 lumbar DRG of a Sema3A^−/−^ embryo cross section are shown (anti-neurofilament immunostaining). a, example of DRG that exhibits three abnormal axon bundles. b, example of DRG that exhibits single axon error. **G:** Quantification of abnormal axon bundles – Absence of BAX results in accumulation of errors between E13.5 and E15.5. Massive error elimination is apparent in both genetic backgrounds at E17.5 (E13.5 P = 0.0348, E15.5 P = 0.0069, and E17.5 P = 0.0404). **H:** Quantification of single axon errors – No accumulation of single axon errors is observed between E13.5 and E15.5 in the Sema3A^−/−^:BAX^−/−^ genotype. Single axon errors are fully eliminated by E17.5 in the Sema3A^−/−^ embryos, while Sema3A^−/−^:BAX^−/−^ embryos still exhibit low but detectable levels of errors (n = 30 DRG per genotype at each of the three embryonic ages, E13.5 P>0.05, E15.5 P>0.05, and E17.5 P<0.05 ).

The vertebrate nervous system undergoes massive cell death during development, with a loss of approximately half the neurons [6]. In the DRG the loss of neurons has been shown to result from apoptosis, peaking between E12–E14 [4], [7], within the same time frame as the correction events described above. We therefore tested whether this naturally occurring apoptosis is responsible for the elimination of axon errors. To block apoptosis during DRG development we utilized BAX (a Bcl-2-related protein) null mice, in which no apoptosis is detected in the developing DRG [7]. This approach has been used successfully in numerous studies over the last decade in order to monitor axon guidance in the absence of factors controlling both survival and guidance, such as TrkA or NGF [8], [9], [10], [11], [12]. In all these studies the absence of BAX blocked apoptosis, the number of neurons in the DRGs increased to about 150% over wild type, even in the absence of critical survival factors, and axon fate was mapped. We crossed BAX mutant mice with Sema3A mutant mice in order to generate embryos in which axon errors are detectable, while BAX-dependent cell-death is blocked (experimental design, Figure 1C). Our results show that axon error corrections are not blocked by apoptosis. In addition, we found that in parallel to the elimination of axon errors, there is extensive cell loss even in the absence of BAX. We therefore suggest that non-apoptotic cell death is likely to be a key mechanism for correcting axon guidance errors.

## Materials and Methods

### Animals

BAX and Sema3A null mutant mice were used as previously described [13]. In all experiments, a heterozygous breeding strategy (Sema3A^+/−^:BAX^+/−^ over Sema3A^+/−^:BAX^+/−^) were used to obtain wild-type, Sema3A^+/+^:BAX^−/−^, Sema3A^−/−^:BAX^+/+^, Sema3A^−/−^:BAX^−/−^, Sema3A^+/−^:BAX^−/−^, and Sema3A^+/−^ :BAX^+/+^ animals. Animal handling adhered strictly to national and institutional guidelines for animal research and was approved by the Ethics Committee of the Hebrew University.

### Neuronal Cell Count

Neuronal number estimates were made using “the optical dissector method” previously described [14].

L5 DRG (a total of two per embryo) of P0 mice were dissected as a whole, fixed and stained with anti-neurofilament antibody 2H3 to visualize neuronal somas and DAPI to visualize nuclei. Whole-mount immunostaining was performed as described [13].

Serial optical Z-sections (5 µm) of the entire ganglion were imaged using a laser-scanning confocal microscope (Olympus IX70). Two ganglia per embryo were imaged based on the integrity of the tissue and the staining quality. Adjacent optical sections were used as a dissector where the first optical section was the ‘sample’ section and the next section functioned as the ‘look up’ section. Nuclei of neurofilament-positive cells were counted manually if they did not appear in the ‘look up’ section, thereby eliminating over- or underestimation of neuronal counts (ImageJ). Cells were counted by an observer who was blind to the genotype of the embryos tested.

In each ganglion, every 5th section (25 µm intervals) was used as a sample section and the average count per section was multiplied by the total section number to give the estimate of the total neuronal number per ganglion.

Only litters with a wild-type genotype as a reference were used. An average of total neuronal number per ganglion of each embryo was determined. Results represent the average total neuronal number per ganglion of each genotype (a total of seven litters with seven wild-type embryos, four Sema3A^+/+^:BAX^−/−^ embryos, three Sema3A^−/−^:BAX^−/−^ embryos and five Sema3A^−/−^:Bax^+/+^ embryos).

### Quantification of Lateral Projection Axon Defects

Quantification of axon defects was done as previously described [4]. Briefly, embryos from the indicated time points and genotypes were fixed in 4% paraformaldehyde in PBS (pH 7.0), cryo-preserved in 30% sucrose and embedded in OCT. Embryos were cryo-sectioned transversely through the hindlimb region. Entire L1 to L5 DRGs were serially sectioned at 20 µm, stained with anti-neurofilament antibody 2H3, and analyzed for abnormal lateral axon projections (see diagram in Figure 1A). Only sections with both a clear DRG profile and a lateral projection from the DRG were scored as abnormal. When abnormal axons appeared in the same general location on adjacent serial sections of tissue, the axons were scored as one lateral projection defect.

Immunohistochemistry for 2H3 was performed as previously described [15]. All pictures presented were taken using Olympus BX51, 100×, NA1.3.

### Statistical Analysis

**Figure 2:**
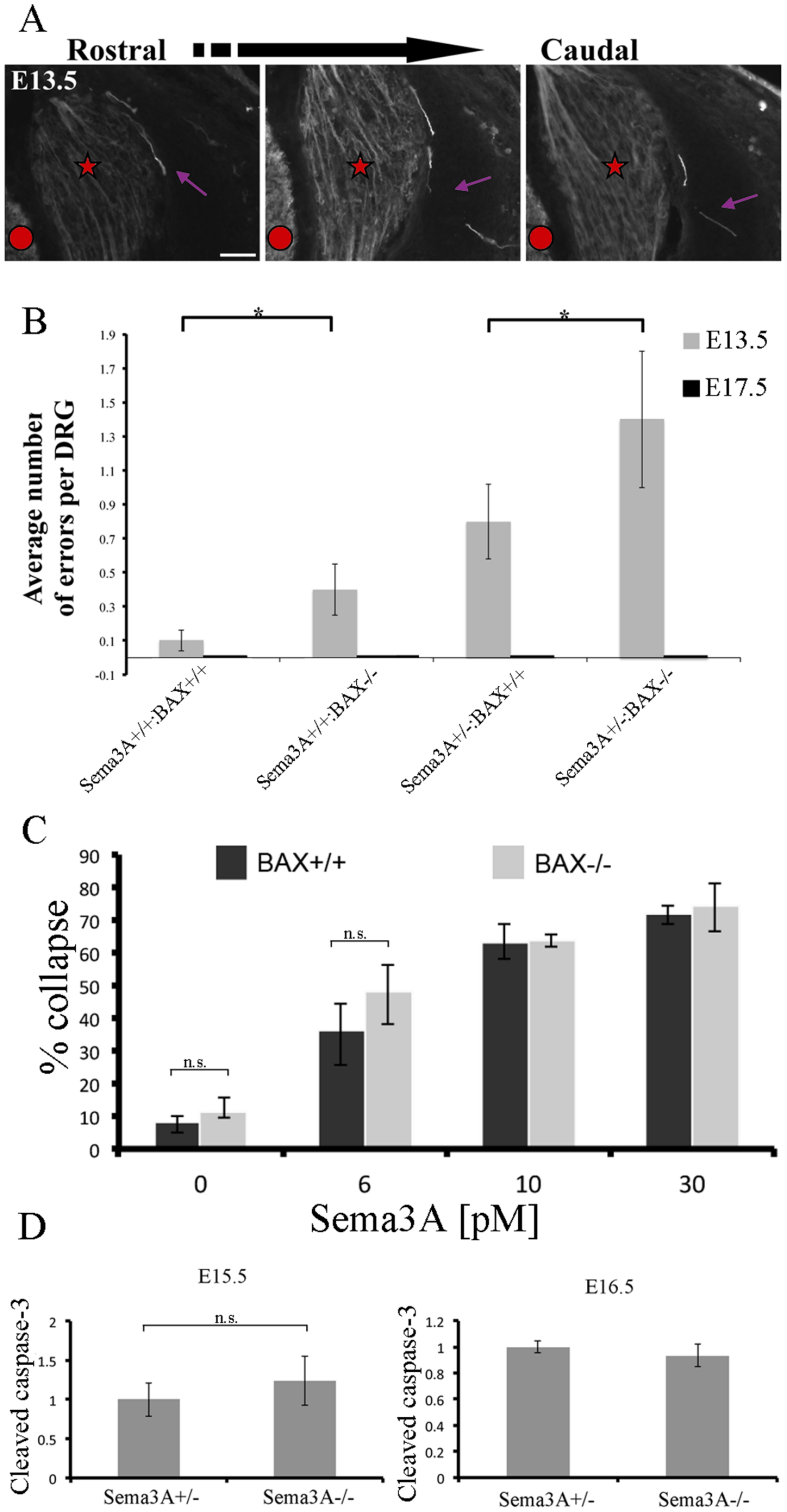
Increased levels of axon guidance errors in BAX null and heterozygote Sema3A:BAX null mice. **A:** Representative image of errors observed in DRG from Sema3A^+/+^:BAX^−/−^ mice. Three adjacent cross sections of E13.5 DRG are shown. Most of the observed errors in the indicated genotypes were single axons or small bundles. Note the tortuous path an abnormal axon takes in and out of the plane of view. Scale bar 50 µm. **B:** Analysis of error occurrence in the indicated genotypes at E13.5. Increase in error frequency is detected in BAX null background, indicating cell-death involvement in both wild-type and Sem3A^+/−^ mice (n = 30 DRG per genotype (Wild type versus Sema3A^+/+^:BAX^−/−^ P = 0.0416, Sema3A^+/−^ :BAX^+/+^ versus Sema3A^+/−^:BAX^−/−^ P = 0.0006). **C:** DRG neurons from BAX^−/−^ and BAX^+/+^ mice are equally responsive to Sema3A-induced growth cone collapse. DRG explants from E12.5 embryos (BAX^+/+^ and BAX^−/−^ littermates) were cultured in the presence of 10 ng/ml NGF for 20 h, at which time neurons were treated with 0, 6, 7.5, 10, 15 or 30 pM Sema3A. After an additional incubation period of 40 min with or without Sema3A, the explants were fixed and stained with rhodamine phalloidin. Quantification of the growth cone collapse results is shown. Results represent the mean +/− S.E.M. of three independent experiments. None of the Sema3A concentrations induced statistically significant difference in collapse levels between BAX^+/+^ and BAX^−/−^ groups, P>0.05. **D:** Activation of caspase-3 is not significantly changed in Sema3A null mice. For each embryo proteins were extracted from E15.5 (upper panel) and E16.5 (lower panel) DRGs from lumbar and thoracic levels (at least three embryos of each genotype were used). Relative changes in caspase-3 activation levels were measured by western blot analysis using an activated caspase-3-specific antibody (Cell Signaling Technology). To determine protein levels each membrane was re-blotted for actin. Quantification of band intensity was obtained using scanning densitometry (Quantity One, BioRad) of three blots representing three different experiments. Results were normalized to actin. The average normalized result of the wild-type embryos at each age was defined as 1. At E15.5 the difference between Sema3A null mice and wild-type littermates is not statistically significant (P>0.05).

**Figure 3:**
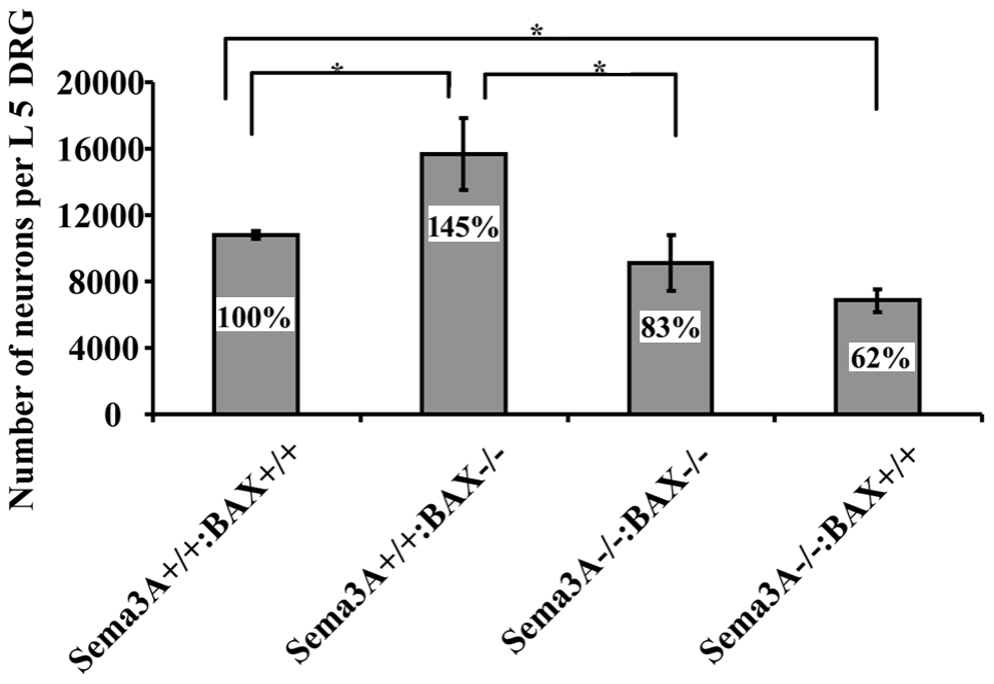
Reduction in DRG neuronal numbers following error correction. Graph representing estimates of DRG neuronal total number. L5 DRG of P0 mice were used as an end point of the error correction period. A significant increase in neuron number as compared to wild-type mice is observed in the Sema3A^+/+^:BAX^−/−^ embryos as a result of apoptosis inactivation (P = 0.0041). A significant decrease in neuron number is observed in the Sema3A^−/−^:BAX^−/−^ embryos as compared to Sema3A^+/+^: BAX^−/−^, which correlates with error correction (P = 0.0169). A pronounced and significant decrease in neuron number is also observed in the Sema3A^−/−^: BAX^+/+^ embryos as compared to wild-type mice (P = 0.0017).(n = 14 wt DRG, n = 8 Sema3A^+/+^:BAX^−/−^ DRG, n = 6 Sema3A^−/−^:BAX^−/−^ DRG and n = 10 Sema3A^−/−^:Bax^+/+^ DRG).

The Mann–Whitney *U* test was used to test the significance of differences in abnormal projections (Figs. 1 and 2) or in DRG neuronal number (Fig. 3). Comparison of different genotypes was done using the average neuron number per DRG of each embryo (two DRG per embryo for neuronal counts and ten per embryo for abnormal projections, three to seven embryos per genotype of seven litters). Symbols are as follows: *P≤0.05, **P≤0.001, ***P≤0.0001.

## Results

### Elimination of Abnormal Axon Projection is not Blocked in the Absence of BAX

DRG on the rostral side begin to develop earlier than those on the more caudal side. Therefore, when comparing DRGs during stages of development it is critical to follow the same rostral/caudal levels. In our study we chose to focus on the lumbar levels since most previous studies that examined axon errors or neuronal numbers focused on the lumbar region.

Lateral projection defects (axons and axon bundles crossing abnormally through the pre-cartilage primordial) of lumbar DRG axons in Sema3A−/−:BAX+/+ and Sema3A^−/−^:BAX^−/−^ littermates were examined at E13.5, E15.5 and E17.5 (see illustration and experimental design outline in Fig. 1A, B). DRG axons were visualized by immunohistochemistry using anti-neurofilament antibodies (Fig. 1C, D). At E13.5 we found similarly high numbers of abnormal axon projections in both genotypes (6.57±0.56 errors per DRG in Sema3A^−/−^:BAX^+/+^ and 5.58±0.87 errors per DRG in Sema3A^−/−^:BAX^−/−^). By E15.5, while the number of errors in Sema3A^−/−^:BAX^+/+^ is unchanged (6.61±0.79 errors per DRG), in Sema3A^−/−^:BAX^−/−^ we observed a doubling of error number (11.16±1.59 errors per DRG, Fig. 1E).

At E17.5 the number of errors is significantly reduced in both genotypes. In Sema3A^−/−^:BAX^+/+^ axon errors are almost completely eliminated (0.63±0.61 errors per DRG, which is a 91% reduction relative to the highest level of errors observed at E13.5 for this genotype). This result is consistent with our previous study [4], although the correction in the present study is delayed, probably reflecting a difference in genetic background from our previous study. The number of errors in Sema3A^−/−^:BAX^−/−^ is reduced, albeit to a lesser degree (3.91±0.79 errors per DRG, a 65% reduction relative to the highest level of errors observed at E15.5 for this genotype, Figure 1E). Thus, axon errors are corrected with and without active BAX (with blocked apoptosis).

Two types of axon errors are detected: axon bundles (for example see Fig. 1Fa) and single axons (for example see Fig. 1Fb). In addition to the quantification of total errors shown in Figure 1E, we also carried out a separate quantification of these two types of axon errors morphologies (Fig. 1G, 1H). In general, the results are similar to the quantifications shown in Figure 1E.

### Increased Levels of Axon Guidance Errors are Detected in Sema3A^+/+^:BAX^−/−^ and Sema3A^+/−^:BAX^−/−^ Mice

To get a broader view of the formation and elimination of axon errors we tested other genotypes obtained by crossing double heterozygote mutants. We tested the degree of lateral projection defects of lumbar DRG axons in wild-type, Sema3A^+/+^:BAX^−/−^, Sema3A^+/−^:BAX^−/−^ and Sema3A^+/−^:BAX^+/+^ littermates at E13.5 and E17.5 (Fig. 2). Most of the observed errors in the indicated genotypes were single axons or small bundles, as shown in a representative image of errors observed in DRG from Sema3A^+/+^:BAX^−/−^ mice (Fig. 2A). In DRG of wild-type mice we detected 0.1±0.06 axon errors per DRG. Interestingly, in Sema3A^+/+^:BAX^−/−^ we detected a small increase in axon errors to 0.4±0.15 errors per DRG. In Sema3A^+/−^:BAX^+/+^ the axon error rate was 0.8±0.22 errors per DRG. Finally, in Sema3A^+/−^ :BAX^−/−^ we detected 1.4±0.46 errors per DRG. Therefore, the rate of errors increased in the absence of BAX. At E17.5 no axon errors were detected in any of the different genotypes (Fig. 2B).

Thus, on BAX null background there is an increased frequency of axon errors both in Sema3A^+/+^, Sema3A^+/−^ or Sema3A^−/−^. What can explain such an effect? One possibility is that a mutation in BAX affects the axon guidance process itself (not the axon error correction mechanism), however two lines of evidence make this possibility unlikely. First, there is a very minor increase in the levels of abnormal projections in mice in which only BAX (Sema3A^+/+^ background) is mutated (0.1 and 0.4 axon errors per DRG in wild-type and Sema3A^+/+^:BAX^−/−^ mice, respectively; see Fig. 2B). Second, growth cones from BAX null mice show no change in their sensitivity to Sema3A-induced growth cone collapse (Fig. 2C).

The difference as a result of BAX inhibition is between Sema3A^−/−^:BAX^+/+^ and Sema3A^−/−^:BAX^−/−^ in E15.5 might indicate a partial role in error correction (Fig. 1E). We therefore searched for evidence of increased apoptosis in Sema3A^−/−^ :BAX^+/+^ mice at this developmental stage (E15.5–E16.5). To this end we compared the levels of cleaved caspase 3 between Sema3A^+/+^:BAX^+/+^ and Sema3A^−/−^:BAX^+/+^ mice at both E15.5 and E16.5. No significant increase in cleaved caspase 3 was detected (Fig. 2D). Thus although one cannot exclude the possibly that apoptosis play a role in axon error correction, we did not find evidence to support this possibility.

### Reduction in DRG Neuronal Numbers Following Error Correction

The results thus far demonstrate that guidance error correction occurs even in the absence of BAX activity, raising the possibility that a BAX-independent non-apoptotic cell death mechanism might be involved. Unfortunately, the lack of reliable tools to evaluate other non-apoptotic, programmed cell death *in vivo* (e.g., autophagy, necroptosis, etc.) did not allow direct testing of this possibility. We therefore carried out direct counts of all DRG neurons to assess the involvement of cell death *per se* in the correction mechanism. In a previous study, Haupt *et al.* found no significant difference in the number of DRG neurons of wild-type and Sema3A mutant mice at brachial, thoracic or lumbar DRG levels at E15.5 [16]. Our observations indicate that the critical time frame for error correction in Sema3A−/− embryos is between E15.5 and P0 (by birth, very few axon errors are detected). We therefore decided to count the number of DRG neurons in newborns at the endpoint of the correction process. We counted the total number of neurons in L5 DRG from wild-type, Sema3A^+/+^:BAX^−/−^, Sema3A^−/−^:BAX^−/−^ and Sema3A^−/−^ :BAX^+/+^ mutant mice (Fig. 3). The average number of neurons in wild-type DRG was 10,779 (defined as 100%). Consistent with previous studies, the average number of neurons in Sema3A^+/+^ :BAX^−/−^ was 15,668 (145% of wild type). This increase in neuron number reflects a blockage of BAX-dependent apoptosis [8], [17].

In Sema3A^−/−^:BAX^−/−^ the average number of neurons was reduced to 9,128 neurons (83% of wild type, or 58% of Sema3A^+/+^:BAX^−/−^), indicating that extensive cell death occurs in the absence of BAX and in association with path-finding error correction. The average number of DRG neurons in Sema3A^−/−^:BAX^+/+^ was only 6,860 (62% of wild type). This reduction in neuron number is associated with error correction and is likely due to the activity of BAX-independent cell-death mechanisms.

## Discussion

We have previously shown that extensive axon guidance abnormalities are largely eliminated during embryonic development by an unknown mechanism [4]. Our current study shows that guidance error correction is not the result of apoptosis, but that another form of cell death is likely the mechanism responsible for this phenomenon. We further observed that neuronal deficits arise from this abnormal projection correction.

### Neuronal Cell Death in Sema3A Null Mice is Secondary to Axon Guidance Error Correction via a Non-apoptotic Mechanism

We found a reduction in postnatal neuronal cell number in both Sema3A^−/−^:BAX^+/+^ and Sema3A^−/−^:BAX^−/−^ mice. The possibility that this cell loss reflects a novel survival-promoting role Sema3A is highly unlikely, since it’s specific co-receptor Nrp1 is not expressed in DRG neurons during the critical period examined [16], [18]. Moreover, *in vitro* studies have reported that Sema3A functions as a death-inducing molecule for embryonic DRG neurons [19], [20]. Thus, the reduction in postnatal neuronal numbers in Sema3A null mice is most likely an indirect result of guidance error corrections occurring between E15.5–P0. This correction mechanism is not apoptosis dependent, since it occurs even in the absence of BAX.

### How can BAX Inhibition Increase Axon Guidance Errors?

Intriguingly, there is actually an increase in early axon guidance errors in the absence of BAX in the embryonic period before error correction, for example at E17.5 the number of errors in Sema3A^−/−^:BAX^−/−^ mice is higher than in Sema3A^−/−^ :BAX^+/+^ mice, although in both genotypes the number of errors is reduced proportionally (Figure 1, panel E, G, H). Moreover, in BAX-only null mice we see a small increase in axon guidance errors compared to wild-type mice. BAX-dependent apoptosis cannot explain this result since we found no indication of a significant increase in apoptosis in Sema3A^−/−^:BAX^+/+^ mice at either E12.5 [13], E15.5 [16] or E15.5–16.5 (Figure 2D). The possibility that BAX has a direct role in axon guidance is also unlikely because BAX null DRG do not exhibit any change in growth cone collapse responsiveness for Sema3A. Most likely, axon guidance errors accumulate in BAX null mice as a consequence of the increased number of DRG neurons during early development, due to inhibition of the naturally occurring cell death [8], [17]. In the absence of Sema3A there is a certain probability of a neuron making an axon guidance error. If this probability is not changed in the absence of BAX, the increased number of neurons due to the lack of apoptosis simply ensures that more neurons are likely to make guidance errors.

### Cell Death is Likely the Mechanism Responsible for Error Correction

The reduction in DRG neuronal numbers in newborns both in Sema3A^−/−^:BAX^+/+^ mice and in Sema3A^−/−^:BAX^−/−^ mice provides the most compelling indication that non-apoptotic cell death corrects axon guidance errors. A recent study analyzing DRG neuronal numbers using the same Sema3A mutant mice at E13.5 and E15.5 found no difference in neuronal numbers at brachial, thoracic or lumbar levels at this stage of development [16]. Thus, neuronal cell loss starts after E15.5, which correlates nicely with the time window for axon guidance error correction. The DRG cell loss observed in the Sema3A^−/−^:BAX^−/−^ genotype differs from the situation described in other mutant lines on BAX null background. Knockouts of several survival promoting factors have been crossed with BAX nulls to prevent sensory neuron cell death [8], [9], [10], [11], [12], and all those cases the compound mutant lines had comparable neuronal numbers as BAX-only mutants (about 150% more than wild-type mice). How can this dramatic difference between all these mice lines and Sema3A null mice be explained? In all these mutations the tested factors are important for survival and are also involved in axon attraction and growth promotion of sensory axons. Thus, most effects are reduced or slower axon growth with only a few guidance errors (entry into forbidden territories). In contrast, in Sema3A mutant mice numerous guidance errors resulting in entry into forbidden territories are detected. Thus, it is likely that passing through these forbidden territories somehow activates non-apoptotic cell death. In addition to apoptosis, neuronal cells may be eliminated by necroptosis or autophagy [21]. Each one of these mechanisms can lead to cell death without the involvement of BAX and without activation of caspase-3 [22]. Markers for monitoring non-apoptotic cell death *in-vivo* have not been sufficiently developed. Thus, the mechanisms by which neurons are eliminated in Sema3A^−/−^:BAX^+/+^ and Sema3A^−/−^:BAX^−/−^ mice are yet to be explored.

### How can Abnormal Guidance Errors be Recognized by a Cell Death Mechanism?

Approximately half of the PNS-born neurons are eliminated throughout development [6], and most of the neuronal loss has been attributed to limited availability of neurotrophic factors during navigation and at peripheral targets [23]. Moreover, upon arrival at the target site, some neurons switch their neurotrophin dependency to another neurotrophin expressed at the site. Thus, axon deviation from intermediate checkpoints may miss a critical survival factor. An example of such a mechanism has been demonstrated for developing commissural neurons [24]. Alternatively cell death-inducing molecules may eliminate misguided neurons. Indeed, axon guidance molecules such as ephrin-A5 and Sema3A may also induce death under specific circumstances [13], [25]. The unusual trajectory of misguided axons may expose them to death-inducing molecules. In this context, it is interesting to note a recent study that finds neither increase in cell death nor reduction in cell number in Npn-1**^Sema^** ^-^ mutant mice, although many aberrant projections do exist [16]. Npn-1 is the binding receptor unit for Sema3A, 3B and 3C, and may also influence signaling by Sema3E and 3F [26]. A few type 3 semaphorins have already been shown to have the potential to induce cell death [13], [27]. In Sema3A null mice, misguided axons may encounter other death-inducing semaphorins. However, misguided neurons in Npn-1**^Sema^** ^-^ mutant mice will be resistant for death induced by other type 3 semaphorins Thus, in Npn-1**^Sema^** ^-^ neuronal loss may not occur.

### Possible Link between Axon Guidance and Susceptibility for Neurodegenerative Diseases

Finally, our findings suggest that a mutation in axon guidance genes could lead to an underpopulated neuronal system at birth. This situation may be non-symptomatic under normal physiological conditions, but could lead to a predisposition for neuronal loss and de-innervation pathologies (neurodegeneration and diabetes). Recent emerging evidence also points to molecules such as presenilin, which play a pivotal role in neurodegenerative disease, as playing an essential role in axon guidance [28]. It would be interesting to test whether genes mutated in neurodegenerative disease are also responsible for inducing developmental axon guidance errors and neuronal death, therefore predisposing for the disease.

## References

[1] Tosney KW, Landmesser LT (1985) Specificity of early motoneuron growth cone outgrowth in the chick embryo. J Neurosci 5: 2336–2344. 2993541 10.1523/JNEUROSCI.05-09-02336.1985 PMC6565323

[2] Kolodkin AL, Tessier-Lavigne M (2011) Mechanisms and molecules of neuronal wiring: a primer. Cold Spring Harb Perspect Biol 3. 10.1101/cshperspect.a001727 PMC3098670 21123392

[3] Fujisawa H (2004) Discovery of semaphorin receptors, neuropilin and plexin, and their functions in neural development. J Neurobiol 59: 24–33. 15007824 10.1002/neu.10337

[4] White FA, Behar O (2000) The development and subsequent elimination of aberrant peripheral axon projections in Semaphorin3A null mutant mice. Dev Biol 225: 79–86. 10964465 10.1006/dbio.2000.9822

[5] Masuda T, Watanabe K, Sakuma C, Ikenaka K, Ono K, et al (2008) Netrin-1 acts as a repulsive guidance cue for sensory axonal projections toward the spinal cord. J Neurosci 28: 10380–10385. 18842897 10.1523/JNEUROSCI.1926-08.2008 PMC6671023

[6] Oppenheim RW (1991) Cell death during development of the nervous system. Annu Rev Neurosci 14: 453–501. 2031577 10.1146/annurev.ne.14.030191.002321

[7] White FA, Keller-Peck CR, Knudson CM, Korsmeyer SJ, Snider WD (1998) Widespread elimination of naturally occurring neuronal death in Bax-deficient mice. J Neurosci 18: 1428–1439. 9454852 10.1523/JNEUROSCI.18-04-01428.1998 PMC6792725

[8] Patel TD, Jackman A, Rice FL, Kucera J, Snider WD (2000) Development of sensory neurons in the absence of NGF/TrkA signaling in vivo. Neuron 25: 345–357. 10719890 10.1016/s0896-6273(00)80899-5

[9] Hellard D, Brosenitsch T, Fritzsch B, Katz DM (2004) Cranial sensory neuron development in the absence of brain-derived neurotrophic factor in BDNF/Bax double null mice. Dev Biol 275: 34–43. 15464571 10.1016/j.ydbio.2004.07.021

[10] Kuruvilla R, Zweifel LS, Glebova NO, Lonze BE, Valdez G, et al (2004) A neurotrophin signaling cascade coordinates sympathetic neuron development through differential control of TrkA trafficking and retrograde signaling. Cell 118: 243–255. 15260993 10.1016/j.cell.2004.06.021

[11] Wickramasinghe SR, Alvania RS, Ramanan N, Wood JN, Mandai K, et al (2008) Serum response factor mediates NGF-dependent target innervation by embryonic DRG sensory neurons. Neuron 58: 532–545. 18498735 10.1016/j.neuron.2008.03.006 PMC2689374

[12] Genc B, Ozdinler PH, Mendoza AE, Erzurumlu RS (2004) A chemoattractant role for NT-3 in proprioceptive axon guidance. PLoS Biol 2: e403. 15550985 10.1371/journal.pbio.0020403 PMC529315

[13] Ben-Zvi A, Manor O, Schachner M, Yaron A, Tessier-Lavigne M, et al (2008) The Semaphorin receptor PlexinA3 mediates neuronal apoptosis during dorsal root ganglia development. J Neurosci 28: 12427–12432. 19020035 10.1523/JNEUROSCI.3573-08.2008 PMC6671732

[14] West MJ (1999) Stereological methods for estimating the total number of neurons and synapses: issues of precision and bias. Trends Neurosci 22: 51–61. 10092043 10.1016/s0166-2236(98)01362-9

[15] Lerman O, Ben-Zvi A, Yagil Z, Behar O (2007) Semaphorin3A accelerates neuronal polarity in vitro and in its absence the orientation of DRG neuronal polarity in vivo is distorted. Mol Cell Neurosci 36: 222–234. 17728139 10.1016/j.mcn.2007.07.003

[16] Haupt C, Kloos K, Faus-Kessler T, Huber AB (2010) Semaphorin 3A-Neuropilin-1 signaling regulates peripheral axon fasciculation and pathfinding but not developmental cell death patterns. Eur J Neurosci 31: 1164–1172. 20345923 10.1111/j.1460-9568.2010.07154.x

[17] Sun W, Gould TW, Vinsant S, Prevette D, Oppenheim RW (2003) Neuromuscular development after the prevention of naturally occurring neuronal death by Bax deletion. J Neurosci 23: 7298–7310. 12917363 10.1523/JNEUROSCI.23-19-07298.2003 PMC6740454

[18] Kawakami A, Kitsukawa T, Takagi S, Fujisawa H (1996) Developmentally regulated expression of a cell surface protein, neuropilin, in the mouse nervous system. J Neurobiol 29: 1–17. 8748368 10.1002/(SICI)1097-4695(199601)29:1<1::AID-NEU1>3.0.CO;2-F

[19] Ben-Zvi A, Yagil Z, Hagalili Y, Klein H, Lerman O, et al (2006) Semaphorin 3A and neurotrophins: a balance between apoptosis and survival signaling in embryonic DRG neurons. J Neurochem 96: 585–597. 16336628 10.1111/j.1471-4159.2005.03580.x

[20] Gagliardini V, Fankhauser C (1999) Semaphorin III can induce death in sensory neurons. Mol Cell Neurosci 14: 301–316. 10588386 10.1006/mcne.1999.0787

[21] Yuan J, Lipinski M, Degterev A (2003) Diversity in the mechanisms of neuronal cell death. Neuron 40: 401–413. 14556717 10.1016/s0896-6273(03)00601-9

[22] Eisenberg-Lerner A, Bialik S, Simon HU, Kimchi A (2009) Life and death partners: apoptosis, autophagy and the cross-talk between them. Cell Death Differ 16: 966–975. 19325568 10.1038/cdd.2009.33

[23] Huang EJ, Reichardt LF (2001) Neurotrophins: roles in neuronal development and function. Annu Rev Neurosci 24: 677–736. 11520916 10.1146/annurev.neuro.24.1.677 PMC2758233

[24] Wang H, Tessier-Lavigne M (1999) En passant neurotrophic action of an intermediate axonal target in the developing mammalian CNS. Nature 401: 765–769. 10548102 10.1038/44521

[25] Gao PP, Sun CH, Zhou XF, DiCicco-Bloom E, Zhou R (2000) Ephrins stimulate or inhibit neurite outgrowth and survival as a function of neuronal cell type. J Neurosci Res 60: 427–436. 10797545 10.1002/(SICI)1097-4547(20000515)60:4<427::AID-JNR1>3.0.CO;2-D

[26] Sharma A, Verhaagen J, Harvey AR (2012) Receptor complexes for each of the Class 3 Semaphorins. Front Cell Neurosci 6: 28. 22783168 10.3389/fncel.2012.00028 PMC3389612

[27] Tomizawa Y, Sekido Y, Kondo M, Gao B, Yokota J, et al (2001) Inhibition of lung cancer cell growth and induction of apoptosis after reexpression of 3p21.3 candidate tumor suppressor gene SEMA3B. Proc Natl Acad Sci U S A 98: 13954–13959. 11717452 10.1073/pnas.231490898 PMC61148

[28] Bai G, Chivatakarn O, Bonanomi D, Lettieri K, Franco L, et al (2011) Presenilin-dependent receptor processing is required for axon guidance. Cell 144: 106–118. 21215373 10.1016/j.cell.2010.11.053 PMC3034090

